# Seasonal Influenza Hospitalization Incidence Rates Among U.S. Active Component Service Members, 2010–2024

**Published:** 2026-01-05

**Authors:** David R. Sayers, Saixia Ying, Angelia A. Eick-Cost

**Affiliations:** Department of Preventive Medicine and Biostatistics, Uniformed Services University of the Health Sciences, Bethesda, MD: Lt Col Sayers; Armed Forces Health Surveillance Division, Defense Health Agency, Silver Spring, MD: Dr. Ying, Dr. Eick-Cost

## Abstract

Despite a longstanding U.S. Department of Defense (DOD) requirement for seasonal influenza vaccination of active component service members (ACSMs), quantifying the impact of the DOD immunization program is challenging. To measure the burden of severe influenza among this highly immunized ACSM population, this study evaluated seasonal and cumulative seasonal influenza hospitalization rates among ACSMs from 2010 through 2024, stratifying by sex, age group, race and ethnicity, service branch, recruit site, and location (U.S. vs. non-U.S.). In contrast to Centers for Disease Control and Prevention (CDC) U.S. population data, the highest ACSM cumulative seasonal influenza hospitalization rate was in the age group under 25 years (9.3 per 100,000 person-years [p-yrs]) and recruits (70.1 per 100,000 p-yrs). Non-U.S.-based ACSMs had lower influenza hospitalization rates (4.8 per 100,000 p-yrs) compared to ACSMs in the U.S. (8.0 per 100,000 p-yrs). Within the DOD, cumulative seasonal influenza hospitalization rates were highest in the youngest age group, particularly among recruits. This may influence DOD influenza vaccine distribution priority considerations in the future.

Influenza vaccines have been employed by the U.S. Department of Defense (DOD) since the 1940s and have been required annually since the 1950s for active component service members (ACSMs). ^1^ Each year, the DOD's goal is to reach greater than 90% influenza vaccine compliance rates by January 15, a goal that is typically achieved, especially for ACSMs. ^2^ The DOD influenza program is challenged with shipping vaccine across the world in a timely manner. Differences in compliance groups are influenced by how quickly vaccines can be sent and used. Historically, non-U.S. locations have been prioritized for distribution first, while U.S. locations (including training sites) are hierarchized as lower in importance.

Quantifying the impact of the DOD influenza program is challenging, as vaccine effectiveness (VE) calculations through traditional, observational test-negative case control studies typically demonstrate lower VE compared to national data. ^3^ Multiple factors may influence this observed lower VE with the DOD, including diminished antibody response to serial annual vaccinations, waning immunity during the influenza season, and study design limitations (i.e., adequate statistical power). ^4^ Evaluating the burden of severe influenza illness among this highly vaccinated population may serve as a surrogate for vaccine performance.

The U.S. Centers for Disease Control and Prevention (CDC) Influenza Hospitalization Surveillance Network (FluSurv-NET) generates cumulative seasonal influenza hospitalization rates, stratified by age group, to define the national burden of influenza disease. Typically, the highest rates of influenza hospitalizations occur in older adults (>50 years) and young children (0-4 years). ^5^ Cumulative seasonal influenza hospitalization rates help quantify the burden of severe illness, but this has not been summarized previously for U.S. ACSMs. Analyzing DOD cumulative seasonal influenza hospitalization rates allows identification of higher risk ACSM groups and comparisons of the highly immunized military population to national trends.

**What are the new findings?**

Compared to U.S. national data, in which adult seasonal influenza hospitalization rates increase with age, the highest cumulative hospitalization rate among active component service members occurred in the youngest age group, those younger than age 25 years, especially in recruit settings.

**What is the impact on readiness and force health protection?**

Lower cumulative rates of seasonal influenza hospitalization in older age groups of active component service members help quantify the impacts of the longstanding DOD vaccination requirement for influenza. The higher burden of hospitalization among recruits offers DOD vaccine distribution priority considerations in the future.

The objectives of this study were to evaluate the cumulative seasonal influenza hospitalization rates of ACSMs by sex, age group, race and ethnicity, service branch, recruit site, and location (U.S. vs. non-U.S.). ACSM seasonal influenza hospitalization rates were also compared to CDC age group rates.

## Methods

The population included all U.S. ACSMs during each influenza season, defined as September 1 through April 30, from the 2010-2011 through 2023-2024 seasons. Data from the Defense Medical Surveillance System (DMSS) and standardized laboratory data provided by the Defense Centers for Public Health–Portsmouth were utilized for the analysis.

Influenza hospitalizations were defined as 1 hospitalization with any of the defining diagnoses of influenza in the first or second diagnostic position (International Classification of Diseases, 10th Revision [ICD-10] codes J09-J11, International Classification of Diseases, 9th Revision [ICD-9] codes 487-488) or laboratory-confirmed influenza-positive result (rapid antigen, RT-PCR, or culture influenza assay) with an indication that the individual was hospitalized. All hospitalizations meeting the inclusion criteria were included in the analysis. There were no exclusions. The incidence date was defined as the first date of hospitalization. An individual could be an incident case only once per influenza season.

For each influenza season, individual person-time began on September 1 or entry into active component service (whichever came last) and ended either April 30, last date in active component service, or incidence date for the hospitalization (whichever came first). Seasonal influenza hospitalization incidence rates (IRs) were calculated as the number of incident influenza hospitalizations divided by the number of person-years (p-yrs) for the season multiplied by 100,000. Incidence rates were calculated overall and stratified by sex, age group, race and ethnicity, service branch, recruit status, and location. Cumulative IRs were also calculated by combining data for the entire surveillance period. Comparisons were made to general U.S. age-stratified influenza hospitalization rates using the CDC Influenza Hospitalization Surveillance Network (FluSurv-NET) data. ^5^

## Results

**Table 1** describes the total cumulative seasonal influenza hospitalizations among ACSMs from 2010 through 2024, stratified by sex, age group, race and ethnicity, service branch, recruit status and location (U.S. vs. non-U.S.). The overall cumulative influenza hospitalization rate was 7.4 per 100,000 p-yrs, with the highest rate among recruits (70.1 per 100,000 p-yrs). Higher hospitalization rates were observed in the youngest age group (<25 years; 9.3 per 100,000 p-yrs), women (9.7 per 100,000 p-yrs), Marine Corps members (13.9 per 100,000 p-yrs), and individuals located in the U.S. (8.0 per 100,000 p-yrs).

**TABLE 1.** Cumulative Seasonal Incidence of Influenza Hospitalizations, by Demographic Characteristics, U.S. Active Component Service Members, 2010–2024

Characteristics	Cases	Person-Time	Incidence Rate ^a^
	No.	Person-Years	
All	1,039	14,066,193	7.4
Sex			
Male	820	11,812,456	6.9
Female	219	2,253,737	9.7
Age, *y*			
<25	499	5,348,026	9.3
25–29	170	3,337,557	5.1
30–39	242	3,930,877	6.2
40+	128	1,449,733	8.8
Race and ethnicity			
White, non-Hispanic	542	8,044,365	6.7
Black, non-Hispanic	195	2,210,087	8.8
Hispanic	176	2,165,838	8.1
Other, unknown	126	1,645,903	7.7
Service branch			
Army	418	5,064,269	8.3
Navy	171	3,351,236	5.1
Air Force	163	3,328,304	4.9
Marine Corps	265	1,907,678	13.9
Coast Guard	22	414,706	5.3
Recruit			
No	811	13,741,025	5.9
Yes	228	325,168	70.1
Location			
U.S.	901	11,210,960	8.0
Outside U.S.	138	2,855,233	4.8

Abbreviations: No., number; *y* , years.

a Rate per 100,000 person-years

**FIGURE 1.**
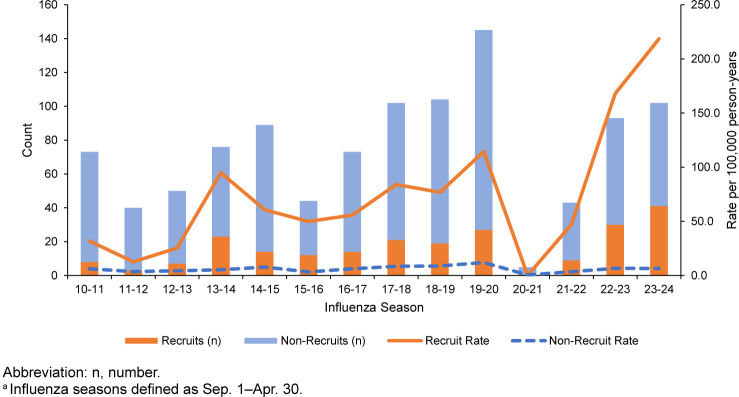
Counts and Incidence Rates of Influenza Hospitalizations, by Recruit Status and Influenza Season ^a^ , U.S. Active Component Service Members, 2010–2024

Seasonal counts and incidence rates of influenza hospitalizations with stratification by recruit status are shown in **Figure 1** . Overall counts varied by annual influenza season, with the largest number of influenza hospitalizations (n=145) during the 2019-2020 season. Counts and rates dropped significantly during the 2020-2021 season, coinciding with the COVID-19 pandemic. The largest number (41) of recruit influenza hospitalizations occurred during the 2023-2024 influenza season. Except for the seasons affected by the COVID-19 pandemic, incidence rates of influenza hospitalizations among recruits trended upwards during the surveillance period, with the highest rate (IR 218.5 per 100,000 p-yrs) observed during the 2023-2024 season.

**Table 2** shows the influenza hospitalization counts and rates for recruits, stratified by age group, sex, race and ethnicity, and service branch. Among recruits, higher cumulative seasonal influenza hospitalization rates occurred in ages younger than 25 years (71.9 per 100,000 p-yrs), men (76.3 per 100,000 p-yrs), and Marine Corps members (178.7 per 100,000 p-yrs).

**TABLE 2.** Cumulative Seasonal Incidence of Influenza Hospitalizations Among Recruits, by Demographic Characteristics, U.S. Active Component Service Members, 2010–2024

Characteristics	Cases	Person-Time	Incidence Rate ^a^
	No.	Person-Years	
All	228	325,168	70.1
Sex			
Male	206	270,075	76.3
Female	22	55,093	39.9
Age, *y*			
<25	209	290,650	71.9
25–29	17	26,754	63.5
30–39	2	7,620	26.2
40+	0	145	0.0
Race and ethnicity			
White, non-Hispanic	129	171,255	75.3
Black, non-Hispanic	33	56,567	58.3
Hispanic	41	64,248	63.8
Other, unknown	25	33,098	75.5
Service branch			
Army	60	123,773	48.5
Navy	4	61,729	6.5
Air Force	15	54,548	27.5
Marine Corps	143	80,022	178.7
Coast Guard	6	5,096	117.7

Abbreviations: No., number; *y* , years.

a Rate per 100,000 person-years

**FIGURE 2.**
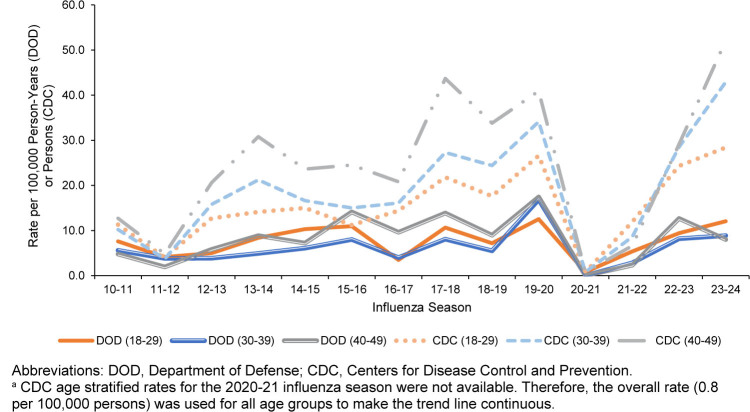
Comparison of U.S. Department of Defense and U.S. Centers for Disease Control and Prevention ^a^ Data for Incidence Rates of Influenza Hospitalizations, by Influenza Season, 2010–2024

**Figure 2** compares seasonal influenza hospitalization rates for ACSMs to CDC age groups. Seasonal influenza hospitalization rates were lower among ACSMs for all age groups compared to CDC age groups. Whereas CDC hospitalization rates increase with older age groups, the ACSM age groups were more comparable throughout each influenza season. When ACSMs younger than age 30 years were further stratified into younger than age 25 years and ages 25-29 years, the younger than age 25 years group had the highest influenza hospitalization rate among all age groups for over half the annual influenza seasons reported (data not shown).

## Discussion

Cumulative seasonal influenza hospitalization rates help quantify the burden of severe illness in a population. In this study, cumulative seasonal influenza hospitalization rates from 2010 through 2024 reveal higher hospitalization rates among the youngest age group (<25 years) of ACSMs. This is counter to CDC national data in which adult influenza hospitalization rates increase with each age group. Hospitalizations within recruit populations drive this increased risk in the youngest DOD age group and in the Marine Corps. Military trainees have historically been vulnerable to acute respiratory disease due to relative immune compromise from physical, environmental, and psychological stress. ^6^ Multiple studies have reported that recruits have a higher incidence of influenza-like illnesses compared to non-recruits. ^7 , 8^

Age-stratified influenza hospitalization rates from CDC national data were higher than the age-stratified ACSM rates. Influenza immunization has been a requirement for the DOD since the 1950s, with goals to reach at least 90% coverage each season. ^1 , 2^ Influenza vaccine coverage among individuals ages 18-49 years in the general U.S. population ranged from 26.9% to 38.4%, depending on the influenza season, from the 2010-2011 through 2023-2024 seasons. ^9^ This differential vaccine coverage is likely a factor in why influenza hospitalization incidence rates among ACSMs were lower than CDC national data rates and do not increase incrementally with each older age group.

Locations outside the continental U.S. are the priority areas for DOD influenza vaccine distribution; however, the non-U.S. influenza hospitalization rate was lower than the rate for U.S. locations. This may be complicated by service members seeking care outside oversees DOD facilities. Future studies could examine influenza vaccination in DOD locations outside the continental U.S. versus U.S. populations. Regardless, the high influenza hospitalization rates in recruits should influence vaccine priority distribution strategies in the future. Areas of additional study need to evaluate factors associated with hospitalizations in the recruit setting and within the Marine Corps.

This study has several limitations. First, influenza hospitalizations were identified using ICD-10-CM (International Classification of Diseases, 10th Revision, Clinical Modification) billing code data, which is dependent on correct coding during inpatient stay and completeness. Inpatient diagnostic coding is entered by nosologists, however, which should ensure higher coding accuracy. The DMSS also has near-complete capture of all ACSM data, including outsourced data in addition to military hospitals and clinics.

Another limitation is the completeness of the laboratory data. Only laboratory testing requested by a military medical facility is captured in these data. This limitation could lead to an under-estimation of hospitalization rates; however, inclusion of ICD-10-CM hospitalization data should cover this gap. The laboratory data also do not indicate if a hospitalization was specifically for influenza, only that the individual testing positive for influenza was hospitalized, which could over-estimate the number of hospitalizations due to influenza. Data evaluating the influenza vaccine performance could not be determined against type or lineage of circulating virus. The incidence of hospitalization was low, along with a small unvaccinated population; thus, this study did not have adequate power to calculate valid vaccine effectiveness estimates.

Although influenza hospitalizations are relatively rare in this population, likely due to the influenza vaccine requirements for service members, these results identify sub-populations within ACSMs at higher risk for severe influenza infections. DOD policies and vaccine distribution should consider these findings to ensure the health and readiness of U.S. service members.
